# Associated factors for treatment delay in pulmonary tuberculosis in HIV-infected individuals: a nested case-control study

**DOI:** 10.1186/1471-2334-12-208

**Published:** 2012-09-07

**Authors:** Isabella Coimbra, Magda Maruza, Maria de Fátima Pessoa Militão-Albuquerque, Líbia Vilela Moura, George Tadeu Nunes Diniz, Demócrito de Barros Miranda-Filho, Heloísa Ramos Lacerda, Laura Cunha Rodrigues, Ricardo Arraes de Alencar Ximenes

**Affiliations:** 1 Post-graduation program in Tropical Medicine, Universidade Federal de Pernambuco, Rua Antonio Rabelo 245, Madalena, Recife, PE, CEP 50610-110, Brazil; 2 The Ageu Magalhães Research Center, FIOCRUZ, Recife, Pernambuco, Brazil; 3 Department of Clinical Medicine, Universidade de Pernambuco, Recife, Pernambuco, Brazil; 4 London School of Hygiene and Tropical Medicine, London, UK

**Keywords:** HIV, Tuberculosis, Delay

## Abstract

**Background:**

The delay in initiating treatment for tuberculosis (TB) in HIV-infected individuals may lead to the development of a more severe form of the disease, with higher rates of morbidity, mortality and transmissibility. The aim of the present study was to estimate the time interval between the onset of symptoms and initiating treatment for TB in HIV-infected individuals, and to identify the factors associated to this delay.

**Methods:**

A nested case-control study was undertaken within a cohort of HIV-infected individuals, attended at two HIV referral centers, in the state of Pernambuco, Brazil. Delay in initiating treatment for TB was defined as the period of time, in days, which was greater than the median value between the onset of cough and initiating treatment for TB. The study analyzed biological, clinical, socioeconomic, and lifestyle factors as well as those related to HIV and TB infection, potentially associated to delay. The odds ratios were estimated with the respective confidence intervals and *p*-values.

**Results:**

From a cohort of 2365 HIV-infected adults, 274 presented pulmonary TB and of these, 242 participated in the study. Patients were already attending 2 health services at the time they developed a cough (period range: 1 – 552 days), with a median value of 41 days. Factors associated to delay were: systemic symptoms asthenia, chest pain, use of illicit drugs and sputum smear-negative.

**Conclusion:**

The present study indirectly showed the difficulty of diagnosing TB in HIV-infected individuals and indicated the need for a better assessment of asthenia and chest pain as factors that may be present in co-infected patients. It is also necessary to discuss the role played by negative sputum smear results in diagnosing TB/HIV co-infection as well as the need to assess the best approach for drug users with TB/HIV.

## Background

In 2010, around 8.8 million cases of tuberculosis (TB) were reported worldwide, 13% of which were HIV-infected individuals. TB was responsible for the death of around 350,000 people living with HIV [1]. Brazil is amongst 22 countries with the highest levels of TB in the world, and preliminary data for the year 2011 has shown an incidence of 43/100,000 inhabitants and 4600 deaths per year associated to TB [2]. In 2010, AIDS-related deaths in Brazil were registered as 1.5/100,000. In Brazil, TB is the primary cause of death in HIV-infected individuals [2]. In the state of Pernambuco around 18,000 cases of HIV-infected individuals have been reported during the last 30 years, with an incidence in 2010 of 17/100,000 inhabitants. Pernambuco has the second highest death rate from TB in Brazil. Partial data for the year 2011 indicated that of the 4694 reported TB cases in the state, 11% were HIV positive [3].

Early diagnosis of TB, particularly the pulmonary form, is essential in order to initiate treatment and control the disease [4]. In HIV-infected individuals, delay in initiating treatment for TB is an important factor for high morbidity [5], mortality [1] and transmissibility of the disease [6,7], and may also result in the prolonged occupation of hospital beds, both in developing countries and in industrialized countries [8]. It should also be noted that in TB/HIV co-infection, the interaction between *Mycobacterium tuberculosis* and HIV results in more rapid progression of both TB and HIV [9]. One further problem is that a high degree of immunodeficiency may modify the clinical and radiological features of TB [10], thus making diagnosis even more difficult and may lead to death before TB treatment has been initiated [11].

Several studies have addressed the problem of delayed diagnosis and treatment for TB, but only a few with HIV-infected individuals [5,8,12-19]. Of these reports, only the studies by Kramer [8] and Hudson [12] were conducted exclusively with HIV-infected individuals.

Systematic reviews on the subject [20-22] have highlighted the lack of uniformity regarding the definition of delay (especially when related to health services), the characteristics of the populations studied, the sites where studies are conducted and the prevalence of TB and HIV. It may also be observed that HIV infection variables as potential predictors of delay have only been included in a small number of the selected studies [22].

The aim of the present study was to assess the time interval between the onset of symptoms and the initiation of treatment for pulmonary TB in patients living with HIV, in referral centers for the treatment of HIV, and to identify the factors associated with this delay.

## Methods

### Study design and population

A nested case-control study was undertaken within a cohort of HIV-infected individuals, aged 18 years and over, who had initiated treatment for pulmonary TB at two referral centers for HIV/AIDS in the state of Pernambuco, Brazil, during the period from July 2007 to February 2010 at one health center, and from October 2007 to June 2010 in another. Around 70% of all HIV-infected individuals in the state of Pernambuco attend these two centers, which provide both outpatient and inpatient care.

**Figure 1:**
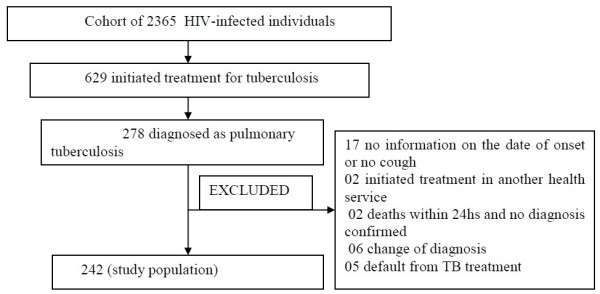
Algorithm for selection of patients for the study of factors associated with delay in initition of treatment for pulmonary tuberculosis in HIV-infected individuals.

Exclusion criteria: (1) patients with no information on the date of onset of cough or no cough; (2) patients who had initiated treatment in another health service; (3) patients for whom TB treatment had been initiated by an attending physician by clinical suspicion, but whose diagnosis had changed during a follow-up period of at least 30 days (after initiating TB treatment), the length of time necessary to observe whether there has been an improvement in the clinical and radiological findings or (4) patients with no laboratory confirmation (smear and culture negative sputum) who were discharged or who defaulted within a period shorter than 30 days after initiating TB treatment. This was a strategy to minimize misclassification (patients mistakenly classified as TB cases) (Figure 1).

### Patient recruitment

Patients attending each service were invited to participate in the study. Those who agreed were interviewed by previously trained health professionals, using standardized questionnaires, after patients had signed the informed consent forms. Additional information was obtained from medical records.

### Definition of terms and variables

Cases of active pulmonary TB were those for whom TB treatment had been initiated by an attending physician through laboratory confirmation by sputum smear and/or sputum culture or clinical suspicion. Cases of disseminated TB with lung involvement, and those with extra-pulmonary TB associated to pulmonary TB, were also defined as pulmonary TB.

**Figure 2:**
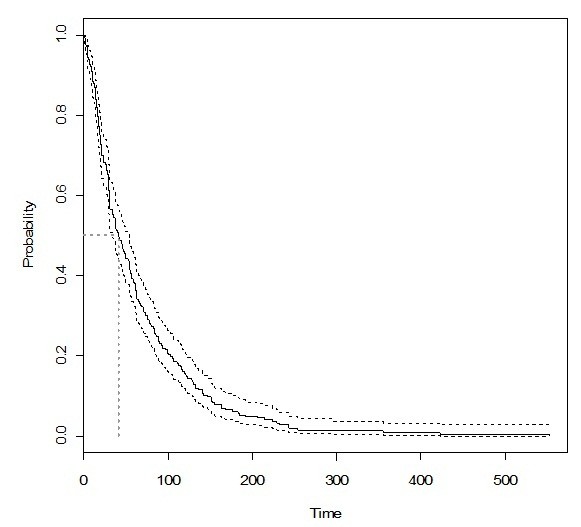
Kaplan-Meier curve for the start of tuberculosis treatment after the onset of symptoms in a cohort of HIV-infected individuals.

There were no studies that could provide a uniform definition of delayed treatment within this group of patients. Thus, to decide the best cut-off point we firstly used the Kaplan-Meier estimator to calculate the probability of starting tuberculosis treatment (Figure 2). Subsequently, the following cut-off points were tested: the median (41 days), 30, 60 and 86 days (values in the third quartile of the distribution curve). Use of drugs was the only variable, which remained in all multivariate models (data not shown). We assumed the median as the cut-off point and delayed TB treatment was defined as the period of time, in days, which exceeded the median value between the onset of cough and the initiation of TB treatment. In the present study, the evaluated delay was considered as a delay related to health services, since all patients were being monitored by these services before the onset of cough.

A study case was defined as a patient who presented a delay in initiating treatment and a control as a patient who did not present a delay in initiating treatment.

For purposes of the analysis, independent variables were grouped into six blocks: biological variables (sex, age (<30; 30-49; ≥50 (years)); clinical variables (fever, weight loss, sweating, asthenia, sputum production, hemoptysis, chest pain, body mass index [BMI]), socioeconomic variables (town of residence, marital status, social support/living with whom, education, employment); variables related to habits and lifestyle (smoking, drinking and illicit drug use); variables related to HIV (opportunistic disease, AIDS, CD4 T-cell count (the CD4 count was measured over a period of less than 4 months, before the start of treatment and was categorized with the intervals: ≤ 50, 51 – 200, 201 – 500, > 500 (cells/mm3)), use of antiretroviral therapy); variables related to TB (initiation of treatment in outpatients or hospital, radiological pattern, past history of TB, contact with person undergoing treatment for TB, sputum smear or sputum culture.

With regard to the variable of alcohol consumption, individuals were considered drinkers if they: drink half a portion of beer (400ml), a glass of wine (250 ml) or spirits (60 ml). Abstainers were considered those who either never drink or drink less than eight units a year, light drinkers as those who drink on no more than two days a week (without exceeding ten units per month), heavy drinkers as those who drink in excess of five units per day at least 3 to 4 times a week, and alcohol dependent if they were undergoing treatment for alcoholism. For analysis, individuals were placed into three categories: abstainer/light drinker (has never drunk or drinks two days a week, but less than 10 drinks/month), moderate/heavy drinker (drinks 3 to 4 days a week with more than 5 drinks/day, and those undergoing treatment for alcoholism).

With regard to smoking, individuals were categorized as: non-smokers (those who have never smoked); ex-smokers (those who stopped smoking at least six months prior to the study) and smokers (those who smoked at inclusion of the study or had stopped smoking for less than six months).

The criteria used to define AIDS were those adopted by the Ministry of Health in Brazil [23].

The variable use of drugs was analyzed in two manners: one, with an independent evaluation of each drug, and the other considering the use of at least one drug (marijuana, cocaine, crack and glue). For the multivariate analysis the variable use of drugs was employed.

The presence of 1, 2 or 3 constitutional symptoms of fever, weight loss or asthenia was assessed as a compound variable, which for purposes of analysis are referred to as systemic symptoms.

Antiretroviral therapy (ART) was defined as a combination of three different antiretroviral drugs, regardless of the number of drug classes used.

### Statistical analysis

The mean and median time interval between the onset of cough and the initiation of treatment for TB were calculated, in days. The magnitude of the association of each variable of the study with treatment delay was measured by odds ratio (OR) and the statistical significance was tested by the confidence interval (CI) and *p* value (Chi-square test or maximum likelihood ratio). The significance level was set at *P* < 0.05.

Since the time elapsed between the onset of symptoms and the first consultation could influence the delay time, the period of time between the onset of symptoms and the first consultation was compared according to the different categories of each variable that remained in the final model, using the Mann–Whitney or Kruskal-Wallis tests.

Double data entry was performed, which were then compared, validated and subsequently corrected. Data entry was performed concurrently with data collection, and the database was managed by SQL 2000 (Microsoft), using GeneXus 7.5. Data were analyzed using the R version 2.10.0.

A multivariate logistic regression model was used in two stages. In the first, a multivariate regression analysis was undertaken in each group, starting with a minimal model and adding one variable at a time. Variables associated to treatment delay where *P* < 0.20 in the univariate analysis were subsequently included in the intragroup logistic regression model, and those associated with a *P* value ≤ 0.05 remained in the model. In a second stage, for the final multivariate model: variables selected in the previous stage were introduced into the final multivariate model (including variables from all groups) and those with a *P* value ≤ 0.05 remained in the final model.

This study is part of the CSV Project 182/06 - Project for clinical and epidemiological study of TB/HIV co-infection in Recife, approved by the Ethics Committee of the Universidade Federal de Pernambuco (registration SISNEP FR-067 159/CAAE 0004.1.172.106-05 / register CEP / CCS / UFPE 254/05).

## Results

From a cohort of 2365 HIV-infected individuals, 629 initiated treatment for TB during the period of study. Of these, 274 had the pulmonary form of the disease and constituted the study population. Of these, 32 were excluded according to the algorithm of Figure 1. Two hundred and forty-two patients participated in this study. The age-range of the study population was 18-67 years, with an average of 38.2 years (SD = 10.09 years), and 69.4% of patients were male.

The time interval between the onset of cough and the initiation of treatment for TB ranged from a minimum of 1 day to a maximum of 552 days, with a median of 41 days (19-85 days, interquartile range). The mean CD4-cell count was 213.9 cells/mm^3^ and a median of 142 cells/ mm^3^ with a range of 1 to 1,254 cells/mm3, and 57.8% were taking ART at the initiation of treatment for TB. A total of 110 patients (45.4%) were living with AIDS, and 30.9% reported using some form of illicit drug. Sputum smear was negative in 40.9% and was not performed by 23.9%. Sputum culture was not performed in 65.1% of the patients, and was positive for 11.8%. No information was available concerning sputum smear and culture for 0.7% and 19,4% respectively, of the study population. It is probable that these patients did not perform these tests.

The results of the univariate analysis of the factors associated to a delay in initiating treatment for TB in HIV-infected individuals are presented in Table 1. The variables that indicated a statistically significant association with delay, in the univariate model were: - clinical variables: weight loss, asthenia, sweating and chest pain; - variables related to habits and lifestyle: use of marijuana, cocaine and crack; - variables related to TB: sputum smear-negative; - variable composed of systemic symptoms: presence of two or three constitutional symptoms (fever and/or weight loss and/or asthenia).

**Table 1:** Univariate analysis of the factors associated with a delay (defined according to the median value (cutoff)) in initiating treatment for tuberculosis in HIV-infected individuals

**Variable**		**Delayed (> 41 days)**		**No delay (≤ 41 days)**		**OR**	**95%- CI**	**p-value**
		**n(120)**	**%**	**n(122)**	**%**			
**BIOLOGICAL**								
**Sex**	**(n = 242)**							
Female		35	29.17	39	31.97	1.00		
Male		85	70.83	83	68.03	1.14	0.66-1.98	0.6365
**Age**	**(n = 242)**							
< 30 years		24	20.00	24	19.67	1.00		
30 – 49 years		80	66.67	79	64.75	1.01	0.53-1.94	0.9695
≥ 50 years		16	13.33	19	15.57	0.84	0.35-2.02	0.6997
**SOCIOECONOMIC**								
**Town of residence**	**(n = 239)**							
Countryside		20	16.81	19	15.83	1.00		
Capital/metropolitan region		99	83.19	101	84.17	0.93	0.47-1.85	0.8387
**Marital status**	**(n = 241)**							
Married		26	21.67	25	20.66	1.00		
Single/separated/widowed		94	78.33	96	79.34	0.94	0.51-1.75	0.8485
**Living with**	**(n = 228)**							
Family/partner		92	81.42	97	84.35	1.00		
Alone		21	18.58	18	15.65	1.23	0.62-2.48	0.5571
**Education**	**(n = 241)**							
Up to 9 years of school		67	56.30	77	63.11	1.00		
9 or more years of school		52	43.70	45	36.89	1.33	0.79-2.23	0.2814
**In employment**	**(n = 241)**							
No		83	69.75	83	68.03	1.00		
Yes		36	30.25	39	31.97	0.92	0.53-1.59	0.7737
**Lifestyle**								
**Drinking**	**(n = 208)**							
Abstainer/light drinker		91	91.00	100	92.59	1.00		
Moderate/heavy		9	9.00	8	7.41	1.24	0.45-3.42	0.6757
**Smoking**	**(n = 241)**							
Never smoked		44	36.97	51	41.80	1.00		
Ex-smoker		49	41.18	51	41.80	1.11	0.63-1.96	0.7076
Smoker		26	21.85	20	16.39	1.51	0.74-3.09	0.2569
**Use of illicit drugs**	**(n = 239)**							
No		70	59.32	94	77.69	1.00		
Yes		48	40.68	27	22.31	2.39	1.37-4.24	**0.0025**
**Use of marijuana**	**(n = 239)**							
Yes		47	39.83	27	22.31	1.00		
No		71	60.17	94	77.69	0.43	0.24-0.76	**0.0038**
**Use of cocaine**	**(n = 239)**							
Yes		19	16.10	9	7.44	1.00		
No		99	83.90	112	92.56	0.42	0.17-0.94	**0.0417**
**Use of crack**	**(n = 239)**							
Yes		23	19.49	7	5.79	1.00		
No		95	80.51	114	94.21	0.25	0.10-0.59	**0.0025**
**Use of shoemaker glue**	**(n = 239)**							
Yes		11	9.32	11	9.09	1.00		
No		107	90.68	110	90.91	0.97	0.40-2.36	0.9507
**Variables**		**(Delayed) > 41 days**		**(No delay) ≤ 41 days**		**OR**	**95%- CI**	**p-value**
		**n**	**%**	**n**	**%**			
**HIV Variables**								
**Opportunistic disease**	**(n = 238)**							
Yes		73	72.12	72	71.96	1.00		
No		45	27.88	48	28.04	0.92	0.55-1.56	0.7682
**AIDS**	**(n = 238)**							
Yes		53	44.92	57	47.50	1.00		
No		65	55.08	63	52.50	1.11	0.67-1.85	0.6893
**CD4 Count**	**(n = 175)**							
≤ 50 cels/mm^3^		27	32.14	23	25.27	1.00		
51 – 200 cels/mm^3^		25	29.76	35	38.46	0.61	0.28-1.29	0.1982
201 – 500 cels/mm^3^		22	26.19	24	26.37	0.78	0.35-1.74	0.5458
>500 cels/mm^3^		10	11.90	9	9.89	0.95	0.33-2.77	0.9189
**Use of ART**	**(n = 240)**							
Yes		67	55.83	73	60.83	1.00		
No		53	44.17	47	39.17	1.23	0.74-2.06	0.4323
**TB VARIABLES**								
**Place where initiated TB treatment**	**(n = 180)**							
Outpatients		42	46.67	42	46.67	1.00		
Inpatients		48	53.33	48	53.33	1.00	0.56-1.80	1.0000
**Standard chest x-ray**	**(n = 220)**							
Atypical		92	85.19	92	82.14	1.00		
Normal		1	0.93	3	2.68	0.33	0.02-2.66	0.3453
Typical		15	13.89	17	15.18	0.88	0.41-1.87	0.7443
**Contact with person undergoing TT TB**	**(n = 239)**							
Yes		81	68.64	84	69.42	1.00		
No		37	31.36	37	30.58	1.04	0.60-1.80	0.8966
**TB contact in the household**	**(n = 75)**							
Yes		15	40.54	10	26.32	1.00		
No		22	59.46	28	73.68	0.52	0.19-1.38	0.1940
**Previous treatment**	**(n = 236)**							
Yes		29	24.58	31	26.27	1.00		
No		89	75.42	87	73.73	1.09	0.61-1.97	0.7650
**Sputum smear**	**(n = 225)**							
Positive		29	26.13	39	34.21	1.00		
Negative		58	52.25	41	35.96	1.90	1.02-3.58	**0.0438**
Not performed		24	21.62	34	29.82	0.95	0.47-1.93	0.8858
**Sputum Culture**	**(n = 195)**							
Positive		14	15.05	9	8.82	1.00		
Negative		27	29.03	18	17.65	0.96	0.34-2.68	0.9447
Not performed		52	55.91	75	73.53	0.45	0.17-1.09	0.0815
**Weight loss**	**(n = 221)**							
No		30	26.55	47	43.52	1.00		
Yes		83	73.45	61	56.48	2.13	1.22-3.78	**0.0086**
**Anemia**	**(n = 169)**							
No		17	19.77	19	22.89	1.00		
Yes		69	80.23	64	77.11	1.20	0.58-2.54	0.6202
**Asthenia**	**(n = 241)**							
No		40	33.33	64	52.89	1.00		
Yes		80	66.67	57	47.11	2.25	1.34-3.80	**0.0023**
**Sweating**	**(n = 233)**							
No		56	47.46	70	60.87	1.00		
Yes		62	52.54	45	39.13	1.72	1.03-2.90	**0.0406**
**Variables**		**Delayed > 41 days**		**No delay ≤ 41 days**		**OR**	**95%- CI**	**p-value**
		**n**	**%**	**n**	**%**			
**Production of Sputum**	**(n = 242)**							
Yes		74	61.67	77	63.11	1.00		
No		46	38.33	45	36.89	1.06	0.63-1.79	0.8161
**Dyspnea**	**(n = 213)**							
No		68	63.55	77	72.64	1.00		
Yes		39	36.45	29	27.36	1.52	0.85-2.74	0.1558
**BMI**	**(n = 211)**							
≥ 18.5		75	72.12	77	71.96	1.00		
< 18.5		29	27.88	30	28.04	0.99	0.54-1.81	0.9803
**Hemoptysis**	**(n = 230)**							
No		104	88.89	102	90.27	1.00		
Yes		13	11.11	11	9.73	1.16	0.50-2.75	0.7330
**Chest Pain**	**(n = 222)**							
No		70	61.40	81	75.00	1.00		
Yes		44	38.60	27	25.00	1.89	1.07-3.38	**0.0309**
**Constitutional Symptoms**	**(n = 242)**							
None		13	10.83	26	21.31	1.00	-	
1*		23	19.17	37	30.33	1.24	0.54-2.94	0.6136
2**		40	33.33	36	29.51	2.22	1.01-5.07	**0.0515**
3***		44	36.67	23	18.85	3.83	1.69-9.04	**0.0016**

OR = Odds Ratio CI = Confidence Interval ART = Antiretroviral Therapy TB = Tuberculosis.

TT TB = TB treatment.

1* asthenia or weight loss or fever.

2* (asthenia and weight loss) or (asthenia and fever) or (weight loss and fever).

3* asthenia and weight loss and fever.

Two multivariate models were run, one introducing the variable asthenia and, the other, introducing the variable systemic symptoms. No statistically significant difference was observed between the two models (p = 1). Variables that remained in the first final model were: asthenia (OR: 1.93, 95%-CI: 1.05-3.59), chest pain (OR: 2.16, 95%-CI: 1.10-4.19), use of illicit drugs (OR: 2.79, 95%-CI: 1.47-5.43), sputum smear-negative (OR: 2.22, 95%-CI: 1.10-4.54). Variables in the second model are presented in Table 2.

**Table 2:** Results of the final multivariate model* of factors associated to a delay in initiating treatment for tuberculosis, including systemic symptoms in HIV-infected individuals

**Variables**	**OR**_**adj**_	**95%- CI**	**p-value**
**Systemic Symptoms**			
None	1.00	-	
1 symptom**	1.25	0.50 – 3.22	0.6330
2 symptoms***	1.56	0.63 – 3.94	0.3345
3 symptoms****	2.41	0.95 – 6.29	**0.0679**
**Use of Drugs**			
No	1.00	-	
Yes	2.56	1.35 – 4.95	**0.0044**
**Chest Pain**			
No	1.00	-	
Yes	2.14	1.13 – 4.16	**0.0214**
**Sputum smear**			
Positive	1.00	-	
Negative	2.23	1.11 – 4.57	**0.0264**
Not performed	1.28	0.57 - 2.87	0.5502

OR = Odds Ratio CI = Confidence Interval.

*Model run with 204 observations.

** asthenia or weight loss or fever, *** (asthenia and weight loss) or (asthenia and fever) or (weight loss and fever),.

**** asthenia and weight loss and fever.

There was no difference in time between the onset of cough and a new consultation according to the categories of the variables that remained in the final multivariate model (Table 3).

**Table 3:** Time to a new consultation after the onset of cough according to the variables that remained in the multivariate model

**Cough 1**^**st **^**consultation (days)**	**N**	**Median**	**Mean**	**SD**	**p-value**
**Systemic Symptoms**					
None	32	30	43,59	7,53	0,8264*
1* symptom	50	41	63,78	11,90	
2* symptoms	70	36	52,01	6,11	
3* symptoms	60	38	65,7	10,30	
**Use of Illicit Drugs**					
Yes	70	35	63,01	9,98	0,6670**
No	139	31	54,5	5,02	
**Chest Pain**					
Yes	64	57	60,88	6,97	0,2620**
No	128	34	56,19	6,31	
**Sputum smear**					
Positive	62	34	59,4	10,34	0,9822*
Negative	91	34	56,18	6,50	
Not performed	44	30	53,64	8,99	

* Kruskal-Wallis test.

** Mann–Whitney.

## Discussion

In the present study, a median of 41 days was encountered between the onset of cough and the initiation of treatment for pulmonary TB in HIV-infected individuals. When this time interval was greater than the median it was assumed that a delay in initiating treatment had taken place and that it was related to the health service.

Comparison of our results with those of others is not straightforward. There is no optimal cut-off point to define delay and the characteristics of the studied populations differ. Our figure (41 days) was higher than those found in studies conducted in a number of countries throughout Asia and Sub-Saharan Africa (ranging from 13-38 days) [8,14,16,17,24-28], where the median was also the criterion to define health service associated treatment delay, however it was lower than those obtained in Gambia [29]. In a systematic review, the mean delay related to health services in countries with low to moderate financial resources was 28.4 days [21]. There is no basis to judge which of these periods would be acceptable, since a few [17,27,29] of the studies evaluated the consequences of the delay on the outcome of TB treatment.

Differences in the population composition regarding the frequency of HIV-infected individuals is also one important factor that may affect comparability between studies. Finnie et al [22] reported that HIV and its relation with a delay in diagnosing and treating TB was assessed in only 20% of the studies selected for their systematic review. The frequency of HIV-infected individuals in several studies ranged from 16.4% to 67% [14-17], some of which were conducted in countries with a high prevalence of TB/HIV co-infection. Since different criteria were used to define treatment delay it was not possible to compare our findings with those of Kramer [12] and Hudson [8], who also focused their studies on HIV-infected patients.

Patients who participated in the present study had regular scheduled consultations before initiating treatment for TB at the two health centers, which are referral centers for treating HIV-infected individuals. It is possible that the differences found in relation to other studies conducted in places with a similar prevalence of TB/HIV co-infection are related to features of the health services and the definition for treatment delay.

The independent factors associated with a delay in the initiation of treatment were: the use of illicit drugs, chest pain, sputum smear-negative and the presence of at least two constitutional symptoms: fever, asthenia and weight loss. There is evidence that intravenous drug users living with HIV, tend to develop TB more than those living with HIV who are not drug users [30,31]. Nevertheless, drug use has been described as a factor associated with the delayed diagnosis of TB [32]. One explanation for this would be the suppression of the cough reflex as well as the patient’s lack of awareness regarding the cough [31]. This same author suggests that the fear of stigma and the emergence of withdrawal symptoms as the patient comes off the drugs, plus the belief held by health professionals that drug users have poor adherence to long-term treatment, are factors that contribute to a delay in diagnosing TB [31]. It is the belief of this study that the introduction of educational programs for health teams would help to facilitate dialogue with these patients, and that close monitoring would contribute to reducing this delay.

Chest pain is one of the symptoms associated with TB in some studies [18,19,33], but no studies have been encountered with an association of diagnostic and treatment delay of TB related to health services. Ngadaya et al [19] identified that chest pain was associated with the delayed diagnosis of TB, in relation to the patient, since he/she does not attribute sufficient attention to the pain as being a symptom of TB. Chest pain can be attributed to several causes, such as diseases of the pleura, cardiovascular diseases and muscular pain. In a study conducted by our group to diagnose pulmonary TB in HIV-infected individuals with sputum smear negative, no association was revealed between the presence of chest pain and the diagnosis of TB (unpublished data). However, it is necessary to evaluate this information with care, since paying insufficient attention this symptom, although it is correct (as it is associated with the diagnosis), it does not mean that health professionals should exclude the diagnosis of TB when pain is present. Nevertheless, we cannot rule out the possibility that an association between chest pain and delay in the initiation of treatment occurred only by chance.

There was an association of a cough with three constitutional symptoms (referred to in the present study as systemic symptoms) with the delayed initiation of treatment for TB in HIV-infected individuals. These symptoms may be connected to clinical features of other HIV-related illnesses, such as pneumocystis pneumonia or pulmonary fungal diseases, bringing about the need to carry out further investigations into patients, so as to perform a differential diagnosis. It should be noted that the time needed to perform additional tests can play an important role in delayed diagnosis and initiation of TB treatment [6].

Cain *et al.*[34] observed in a diagnostic investigation study that the combination of cough with other symptoms (fever or night sweats) increased the sensitivity for diagnosing TB, reaching 93%. However, specificity was low (36%), and thus, the proportion of false positives was high. It is possible that the explanation for findings of this study regarding the presence of constitutional symptoms also explain the findings of these authors. The present study considers that in order to reduce the delay in starting TB treatment in individuals living with HIV, patient surveillance needs to be constant, regardless of the number of potentially TB-associated symptoms, and should be conducted by all health professionals who provide care for HIV-infected patients. The implementation of more rapid diagnostic methods using genetic and semi-automated techniques could also have a positive impact on this problem.

One factor that may limit the interpretation of our findings with regard to clinical symptoms, is that patients in this study were asked about each symptom separately, using a standardized instrument. This fact may have generated a certain degree of disagreement between information obtained by the survey and those obtained by the physician and through medical records.

Sputum smear-negative was found to be associated with a delay in TB treatment, related to health services, both in this study and in a number of others [5,8,16,26,35].

This fact is of great significance since it is the most widely-used method for diagnosing TB. However, it may fail to detect about 50% of cases of patients with TB/HIV coinfection, due to, amongst other factors, paucibacillary sputum [36]. In the present study, sputum smear-negative was observed in 52% of patients with delayed initiation of treatment, and 22% of the group did not perform a smear test. The CD4 t-cell count, the presence of opportunistic infections and use of ART potentially modify the course and the clinical and radiological features of pulmonary TB [10], and could be associated with diagnostic and treatment delay of TB. However, no association between these variables and the delayed initiation of treatment was encountered.

The radiological presentation of TB in HIV-infected individuals often progresses with diffuse pulmonary infiltrates or other atypical presentations of TB, a fact that causes the need for differential diagnosis with other respiratory diseases such as pneumocystis, unlike that encountered in immunocompetent patients with TB [37].

Although it has been reported that the low sensitivity of a chest X-ray may cause a delay in diagnosing TB in HIV-infected individuals [14], this was not confirmed by the present study.

The present study did not find an association between a delay in treatment for TB and some of the factors described in the literature, such as living in the interior of the state [38,39], the time taken to travel to a health center, as well as the distance between home and where the patient is attended [22]. It is probable that the findings of this study are due to the fact that all patients were already being attended by referral services, and also because in many cities in the state, local governments provide free transport for patients.

The characteristics of the health service where the study was conducted - with a multidisciplinary team to provide health care for HIV-infected patients (allowing diagnostic investigation of various HIV associated diseases, tuberculosis being one) and with smear and radiological examinations at the unit itself - suggest a potentially lower delay in diagnosing and treating tuberculosis. In Brazil, studies carried out in health services with lower complexity, in the cities of Recife [15], Victoria [27] and Rio de Janeiro [40] indicated among the factors associated with delay, the difficulty in the diagnostic suspicion [40], limited availability of diagnostic methods [27] and problems related to the internal organization of the health services [15]. *Storla et al*. [20], in a systematic review on this subject, indicated that, regardless of HIV infection, repeated consultations at the same level of care may cause a delay in the diagnosis and treatment of TB.

The characteristics of the population and the complexity of the referral centers involved in this study, should approximate the time delay in the present study to those cited by other Brazilian and international studies. However, some considerations should be taken into account. With the advent of ART, the survival of HIV-infected individuals has increased [41], thus implying a more complex service for a longer period of time. It is possible that the large numbers of patients attended by these services could cause an overload of pent-up demand on diagnostic resources, besides the difficulty involved in rescheduling missed appointments. The reduced use of culture for diagnosing TB should also be considered, and the method used may cause a delay of up to eight weeks in delivering results. Moreover, the need to use more complex and costly diagnostic methods, especially when the sputum smear is negative, may imply a delay in the diagnosis of TB, as reported in a previous study [6].

The present study had the advantage of being developed at two referral centers in the state of Pernambuco, attending around 70% of all HIV-infected individuals in the state. One further advantage is the fact that treatment for TB in Brazil is conducted exclusively within the public health service. Drugs for HIV are also distributed throughout the state system. These facts reduce the risk of selection bias.

One limitation of this study is that the differences in the elapsed time between the onset of symptoms and the first consultation could possibly influence the delay time. However, the comparison made between the mean time from the onset of cough to a fresh consultation, according to the different categories of each variable that remained in the final model, showed no statistically significant difference, thus minimizing the possibility of this being an alternative explanation for the findings of this study.

## Conclusion

Although many studies have addressed the issue of delay in initiating treatment for TB, very few have actually targeted HIV-infected individuals. Further studies are needed within this population, which address different cutoff points and assess the consequences of delayed diagnosis and treatment in the prognosis of TB/HIV. The present study emphasizes, by the nature of factors associated with delay, the difficulty in diagnosing TB within this specific population and points to the need for greater discussion on the role of asthenia and chest pain as factors that may be present in patients with pulmonary TB.

The value of sputum smear-negative for diagnosing these patients needs to be further discussed with the attending physicians, as well as evaluating the best approach to be adopted for drug users.

It is our belief that studies including qualitative methodology, which assess the most important criteria used by health professionals for initiating treatment for TB together with the establishment of quicker methods for diagnosing TB in public health services, such as genetic or semi-quantitative methods, especially in cases with negative sputum smear, may represent a great contribution to reducing this time period, with decreased morbidity and transmissibility.

## Competing interests

The authors declare that they have no competing interests.

## Authors’ contribution

IC, MM, MFPMA, LVM, GTND, DBMF, HRL, LCR, RAAX made substantial contributions to the conception and design of the study. MM, LVM, DBMF, HRL supervised the study. RAAX, GTDN provided statistical support. IC, MM, MFPMA, LVM, GTND, DBMF, HRL, LCR, RAAX contributed to the writing of the manuscript. IC, RAAX, MFPMA, LCR critically revised the manuscript. All authors read and approved the final manuscript.

## Source of funding

This study received support from the Ministério da Saúde/Programa DST/AIDS/UNESCO (CSV 182/06 - Projeto "Estudo Clínico-Epidemiológico da Co- Infecção HIV/Tuberculose em Recife").

## Pre-publication history

The pre-publication history for this paper can be accessed here:

http://www.biomedcentral.com/1471-2334/12/208/prepub
